# PDGF-Stimulated Corneal Keratocyte Motility and Migration Patterns on Aligned Collagen Fibrils Are Inhibited by Decorin

**DOI:** 10.3390/ijms27156829

**Published:** 2026-07-30

**Authors:** Nathaniel S. Tjahjono, Divya Subramanian, Tarik Z. Shihabeddin, Nishtha Tyagi, Marya Zlotnikova, Miguel Miron-Mendoza, Victor D. Varner, W. Matthew Petroll, David W. Schmidtke

**Affiliations:** 1 Department of Bioengineering, University of Texas at Dallas, Richardson, TX 75080, USA; nathaniel.tjahjono@utdallas.edu (N.S.T.); divya.subramanian@utdallas.edu (D.S.); tarik.shihabeddin@utdallas.edu (T.Z.S.); nishtha.tyagi@utdallas.edu (N.T.); marya.zlotnikova@utdallas.edu (M.Z.); vdv@utdallas.edu (V.D.V.); 2 Department of Ophthalmology, University of Texas Southwestern Medical Center, Dallas, TX 75090, USA; miguel.miron@utsouthwestern.edu (M.M.-M.); matthew.petroll@utsouthwestern.edu (W.M.P.); 3 Department of Biomedical Engineering, University of Texas Southwestern Medical Center, Dallas, TX 75390, USA

**Keywords:** platelet-derived growth factor, aligned collagen fibrils, decorin, migration, cornea, keratocytes

## Abstract

Decorin, a proteoglycan shown to inhibit transforming growth factor β1 (TGF-β1) signaling, has been increasingly considered as a potential anti-fibrotic agent to aid in therapies addressing corneal scarring and blindness. Decorin is also known to interact with a wide range of other growth factors, such as platelet-derived growth factor (PDGF), which has been shown to stimulate corneal cell migration to repopulate the decellularized region of the wounded cornea. In other tissues, decorin has been shown to inhibit PDGF-mediated migration and prolong wound healing. However, our understanding of the effect of decorin on PDGF signaling in the corneal stroma environment is limited. Specifically, thus far, there have been no studies investigating the interaction between decorin and PDGF in the context of the uniquely organized fibrillar collagen extracellular matrix (ECM), which provides topographic cues that guide cell migration during wound healing. The purpose of this study is to use an in vitro model of decorin-coated aligned collagen fibrils analogous to the corneal stroma ECM to investigate this interaction. With this in vitro model, we demonstrate that decorin inhibits the response of PDGF-BB-stimulated keratocytes to a 2-dimensional (2D) aligned collagen fibril ECM, reducing cell alignment, motility, PDGF receptor β (PDGFRβ) phosphorylation, and wound closure. We observed that when presented with a decellularized wound region, cells on the decorin-coated substrates migrated more randomly and were less aligned with the underlying aligned collagen fibrils than cells on uncoated fibrils, resulting in slower wound closure. Furthermore, PDGF-BB-stimulated cells on the decorin coating had increased branching and reduced solidity, representing a shift from the elongated, bipolar morphology typically resulting from PDGF-BB treatment towards the stellate, branched keratocyte phenotype. While decorin seems to be an effective agent against TGF-β1-mediated fibrosis, its interaction with PDGF-BB appears to present other challenges to wound healing that should be considered in the development of therapies for healthy corneal wound healing.

## 1. Introduction

The cornea is the transparent, stratified, and outermost layer of the eye that plays a critical role in the function and maintenance of eye health and vision. As the outermost layer of the eye, corneal transparency is a critical feature, and this is maintained through a complex interplay of both structural and compositional extracellular matrix (ECM) components with stromal cells known as keratocytes. Following a wound, the damaged region is repopulated by neighboring keratocytes stimulated with growth factors such as platelet-derived growth factor (PDGF) to migrate towards the wound via the aligned topographical structure of the stromal ECM [1]. Within the wound, keratocytes are further activated by transforming growth factor beta (TGF-β) to produce contractile forces and secrete ECM components to repair the tissue [2]. In some wounds, the cascade of growth factor signaling and cell differentiation can result in alteration of corneal integrity and transparency through abnormal ECM secretion and fibrosis [3].

In addition to growth factors like PDGF and TGF-β, there is an emerging interest in the biophysical and biochemical signals present in the cornea and how they regulate wound healing [1,4,5,6]. One biophysical component of interest is the unique organization of aligned collagen fibrils in the lamellae that make up the stromal ECM and present aligned topographical features to the corneal keratocytes [7,8]. This unique structure has been shown in both in vivo and ex vivo studies to mediate intrastromal cell migration during wound healing, resulting in streams of aligned corneal fibroblasts migrating along the lamellae towards the decellularized wound [1]. Additionally, in vitro studies have shown that substrate alignment (both aligned collagen fibrils and nanoscale grooves) reduces keratocyte differentiation to myofibroblasts in the presence of TGF-β [6,9,10].

Furthermore, studies have shown that other biochemical components of the ECM, such as decorin, can influence the response of cells to the soluble cues and structure of the ECM [4,5,11,12,13]. Decorin is a small leucine-rich repeat proteoglycan secreted and found in the cornea and other tissues that has high affinity and interaction with a wide range of targets. Notably, decorin has been shown to bind and attenuate signaling of TGF-β and has been widely studied as a potential anti-fibrotic therapeutic agent [14,15,16,17,18,19]. While decorin’s anti-fibrotic interaction with TGF-β is generally recognized in cornea and other tissues, much remains unknown about decorin’s interaction with other components of corneal wound healing. For example, some studies have reported that decorin’s effect on cell behavior is dependent on its association with collagen ECM [16,20]. Since cornea stromal ECM is uniquely organized and important to wound healing [1,8], it is likely that association with decorin will modulate the effects of both decorin and ECM structure on cell behavior. We have previously reported a method of fabricating decorin-coated aligned collagen fibrils to study decorin inhibition of TGF-β signaling in corneal cells [10]. In the present study, we use the same model of decorin-coated aligned collagen fibril micropatterns to investigate the effect of decorin on the response of keratocytes to PDGF-BB using morphometric analysis, timelapse imaging, and an in vitro freeze injury model [1,21].

## 2. Results

### 2.1. Cell Alignment in Response to PDGF Is Reduced on Decorin-Coated Aligned Collagen Fibrils

To investigate the effects of decorin on corneal keratocyte behavior in response to PDGF, we first seeded primary corneal keratocytes (NRK) onto decorin-coated and non-coated aligned collagen fibril micropatterns in either basal serum free media or media supplemented with 50 ng/mL of PDGF-BB for 5 days. As shown in Figure 1, NRKs displayed a stellate morphology typical of quiescent keratocytes on both the decorin-coated and non-coated aligned collagen fibrils when cultured in serum-free media. However, when NRKs were cultured for 5 days in PDGF-BB-containing media, the keratocytes adopted an elongated morphology on both the decorin-coated and non-coated fibrils. On the non-coated fibrils, the NRKs were oriented in the direction of the underlying aligned collagen fibrils (at approximately 0°), while on the decorin-coated fibrils, the orientation of the NRKs appeared to be random.

To quantify these qualitative differences in the orientation of NRKs on the different substrates and culture conditions, we first measured the orientation index (OI) of the cells based on their actin cytoskeleton arrangement. NRKs that were cultured in serum-free media on both uncoated and decorin-coated aligned collagen fibrils had OI values less than 0.1, which was characteristic of random orientation (Figure 2). In contrast, when NRKs were cultured in the presence of PDGF-BB on uncoated aligned collagen fibrils there was an elevated orientation index (OI) similar to what was previously reported after 96 h [21,22]. However, PDGF-mediated cytoskeleton alignment with respect to fibril direction was decreased by a factor of two (*p* = 0.0054) on decorin-coated aligned collagen fibrils. Although non-significant, a similar trend was observed in the orientation of individual NRK nuclei.

### 2.2. Cells Move More Slowly in Response to PDGF on Decorin-Coated Aligned Collagen Fibrils

To further investigate the effect of the decorin coating on corneal keratocyte behavior on aligned collagen fibrils, we recorded timelapse imaging of live cell cultures for 48 h (Videos S1–S4). This duration was chosen to observe the initial landing, spreading, and motility of the cells prior to confluence. Cell tracking plots (Figure 3) of individual cell movement demonstrated a wider spread of cell movement in PDGF-BB-treated cells compared to serum-free cultured cells, and a decrease in movement with the addition of the decorin coating in PDGF-BB-containing media. On average, PDGF-BB-treated keratocytes moved a maximum of approximately 80 µm from their initial starting point, which decreased by roughly 30% to a maximum of 57 µm when cultured on decorin-coated collagen fibrils. Similarly, PDGF-BB stimulated keratocytes on decorin-coated collagen fibril moved about 25% slower than without the decorin coating at 3.4 µm per hour compared to 4.6 µm per hour (Figure S1). At the end of 48 h, PDGF-BB treated cells on decorin-coated collagen fibrils saw a 30% decrease in cumulative distance traveled at 174 µm/cell, averaged from three experiments, compared to 248 µm/cell traveled by PDGF-BB-treated cells on uncoated collagen fibrils. However, despite a clear difference in migration speeds with and without PDGF-BB, cells in these experiments were restricted in their movement due to the uniform cell confluence throughout the aligned collagen fibril micropatterns.

### 2.3. Decorin Coating Reduces NRK Migration (Wound Closure) in Response to PDGF

In addition to promoting cell elongation, bipolarization, and motility, PDGF has been shown in the corneal wound healing to play a key role in stimulating keratocyte migration into the injured, decellularized region [23,24,25]. To determine if the addition of the decorin coating impacts PDGF-BB-stimulated migration and repopulation in the presence of a cell density gradient, we created a decellularized region within a confluent layer of keratocytes seeded on aligned collagen fibril micropattern using a freeze injury assay as previously described [21,26]. Samples were then cultured for 5 days with or without the addition of PDGF-BB and imaged every hour via timelapse microscopy (Videos S5–S8). Although the cells did not fully repopulate the wounded region for any treatment condition (Figure 4), we observed that PDGF-BB treatment resulted in greater relative wound closure (~45%) compared to SF conditions (~10%). With the addition of the decorin coating, PDGF-BB treated cell wound closure decreased nearly two-fold to ~25%, consistent with several other migration studies of other cell types involving decorin [27,28]. Displacement of the leading edge of the cells into the wound was also measured to determine and directly compare wound closure dynamics between conditions. While all PDGF-BB treated cultures saw increased wound closure (Figure 5) when compared to SF cultures, measurement of the distance between the initial wound boundary and the leading edge of the migrating cells revealed faster wound closure without the decorin coating (~8 µm/h) versus with the coating (~4 µm/h).

To investigate the effects of PDGF and the decorin coating on individual cell migration, we also tracked cell X and Y coordinates for 120 h after the freeze injury (Figure 6A–D) and calculated their accumulated distance traveled (Figure 6E). Although treatment with PDGF-BB resulted in the greatest cumulative distance traveled as expected, averaging at ~1300 μm, the addition of the decorin coating resulted in a decrease of ~15% to ~1100 μm. Average cell speed was increased with the freeze injury with a higher speed of 11.3 µm per hour without the decorin coating compared to 9.4 µm per hour on aligned collagen fibrils only (Figure S2). To quantify the extent of directed migration into the wound region, we calculated the maximum distance traveled by cells in the direction of the wound and observed less penetration of the wound region in PDGF conditions by cells on the decorin-coated aligned collagen fibril micropatterns (Figure 6F). Furthermore, analysis of the directionality ratio of the cell trajectories demonstrated a loss of persistent migration direction in PDGF-BB-treated cells with the addition of the decorin coating (Figure 7).

### 2.4. Effects of Decorin Coating and PDGF on NRK Morphology

PDGF stimulation of corneal cells is well known to promote cell elongation and extension of dendritic cell processes into the matrix [29,30]. To investigate the effect of the decorin coating on the morphological response to PDGF, we measured the projected cell area, length, branching, and solidity. PDGF-BB treatment resulted in increased area, length, and solidity, and decreased branching. However, with the addition of the decorin coating to PDGF-BB treated cells, branching increased by ~26% and solidity decreased by ~28% to a value similar to cells without PDGF-BB (Figure 8).

### 2.5. Effects of PDGF and Decorin Coating on PDGF Receptor Phosphorylation

To investigate a potential mechanism by which the decorin coating may be modifying the NRK response to PDGF-BB, we examined the possibility that decorin was modulating PDGF signaling by affecting the phosphorylation of the PDGF receptor through immunofluorescent staining. As shown in Figure 9A, immunostaining of NRKs with an antibody against the phosphorylated PDGFRβ receptor showed little to no fluorescent signal when NRKs were cultured in serum free media on aligned collagen fibrils with and without a decorin coating. In contrast, when NRKs were cultured in the media containing PDGF-BB for only 15 min on uncoated aligned collagen fibrils, we observed fluorescent punctate staining across the entire cell surface. We also observed fluorescent staining of the phosphorylated PDGFRβ receptor in NRKs cultured in PDGF-BB on decorin-coated aligned collagen fibrils; however, both the fluorescent intensity and the number of punctae were reduced when compared to uncoated aligned collagen fibrils. To quantify these qualitative observations, we measured the mean fluorescent intensity values over the individual cell bodies. As observed in Figure 9B, the average fluorescent intensity of the phosphorylated PDGFRβ receptor in cells treated with PDGF-BB was significantly higher than those in the absence of PDGF-BB. In addition, it was observed that the presence of the decorin coating on the aligned collagen fibrils reduced the fluorescent intensity by about 25%. These results demonstrate that the decorin coating can modify the phosphorylation of the PDGFRβ receptor in corneal keratocytes.

## 3. Discussion

In addition to the aligned collagen fibril lamellae in the cornea, compositional elements including proteoglycans like biglycan or decorin contribute to the function and maintenance of the corneal tissue. In the case of decorin, several studies have reported its pluripotent affinity for a variety of targets including collagen I [31], integrin α_2_β_1_ [32,33], and soluble growth factors like PDGF and TGF-β1 [34]. In the context of corneal wound healing, the contributions of PDGF and TGF-β1 are well studied [35,36]. Briefly, PDGF is known to stimulate normally quiescent keratocytes into a proliferative and migratory bipolar phenotype, facilitating prompt repopulation of the wounded region [37]. Meanwhile, TGF-β1 secreted by epithelial cells adjacent to the wound stimulates differentiation of keratocytes into fibroblasts and myofibroblasts to secrete new ECM and generate contractile forces to remodel the corneal stroma [38]. In some cases, abnormal ECM and force generation by the transformed cells can result in fibrotic scar formation, known as corneal hazing, which results in partial to total loss of vision [39]. In efforts to understand and prevent corneal hazing, decorin has been demonstrated to play an inhibitory role in TGF-β1 signaling both through the study of decorin gene therapy in mice and the addition of exogenous decorin in vitro [14,18,40]. However, although decorin has also been shown to interact with PDGF signaling in several ways in various systems including smooth muscle [28,40], skin fibroblasts [27], and liver [13], the role of these interactions has not been well explored in the cornea.

PDGF has been previously established with in vitro studies to promote migration of corneal keratocytes along the direction of the aligned collagen fibrils which make up the lamellae of the cornea stroma [21]. This directed migration results in the alignment of the bipolar PDGF-stimulated cells. Previous studies have demonstrated an inhibitory effect of decorin on PDGF signaling in several cell types including liver cells and both arterial and airway smooth muscle cells [13,20,28]. One hallmark of PDGF signaling in corneal stromal cells is migration in aligned tracks along the direction of the underlying lamellar aligned collagen fibril topography [1]. To determine if decorin has an inhibitory effect on this behavior on aligned collagen fibrils, we measured the alignment of NRK treated with PDGF-BB at 50 ng/mL following 5 days of culture on aligned collagen fibrils and observed a lower degree of alignment when the fibrils were coated with decorin.

Since the alignment of PDGF-BB-stimulated corneal cells is closely related to their motility and migration on the underlying aligned collagen fibrils in the cornea, we also recorded timelapse videos of live cell culture for two days and observed a decrease in the cumulative distance traveled by cells on aligned collagen fibrils coated with decorin after treatment with PDGF-BB. Furthermore, in a five-day timelapse of live cell imaging following a freeze injury of corneal cells on aligned collagen fibrils, the addition of the decorin coating resulted in decreases in the amount of wound closure, individual cell migration distances, and persistence of cell migration. The impaired alignment and migration seen in these experiments may be attributed to several possible mechanisms. As suggested by previous studies, decorin may bind PDGF-BB and sequester it to the ECM, preventing the phosphorylation of PDGF receptor as a byproduct [13,41]. Alternatively, studies have also reported that decorin interferes directly with cell motility on collagen I by binding to integrin β1 [32,33,42], which may disrupt normal adhesion to the aligned collagen fibrils. Interestingly, although cell persistence and wound closure were markedly reduced in these experiments, the decorin coating reduced individual cell migration to a lesser degree with the freeze injury compared to without. We expect that this difference is primarily due to the introduction of a cell density gradient via the decellularized region, which increases available space for cells to migrate and provides a driving force for migration. Notably, however, individual cell migration speed was higher than on wound closure (leading edge speed) on decorin-coated collagen particularly. It is possible that the Interaction of decorin with cell adhesion to collagen I, as previously mentioned, may reduce the impact of the aligned collagen fibril topography on the cells, resulting in less directed migration and effective wound closure without severely altering individual cell movement.

Morphometric analysis of actin-stained migrating PDGF-BB-treated cells also revealed changes in cell branching and solidity on the decorin-coated fibrils. Both the increase in number of cell branching extensions and decrease in solidity seem to demonstrate another aspect of decorin’s attenuation of PDGF-BB signaling in reducing cell elongation and bipolarization. However, cell projected area and length remained elevated from serum-free conditions, suggesting that some response to PDGF-BB is still occurring. This increase in cell branching and consequent decrease in solidity suggest a cytoskeletal rearrangement more closely aligned with the more quiescent phenotype typical of non-stimulated keratocytes. As previously remarked, the elongated and bipolar (less branched) morphology of PDGF-BB-stimulated keratocytes has been associated with accelerated migration along the aligned collagen fibrils during corneal wound healing [1]. Thus, a reversion to a more quiescent-like phenotype may indicate that the addition of decorin to the wound-adjacent stroma may result in reduced or inhibited keratocyte activation/transformation, leading to delayed, reduced, or otherwise altered long-term tissue remodeling.

To further explore a possible mechanism through which decorin may regulate the effects of PDGF in NRKs, we assessed the effects of decorin on phosphorylation of PDGFRβ (Figure 9). Previous studies have shown that phosphorylation of the PDGF receptor (PDGFR) is involved in several signaling pathways related to cell differentiation, migration, and proliferation [43]. Some of the signaling pathways that cross-talk or are activated by the PDGF receptor include phosphatidylinositol-3-kinase (PI3K), Phospholipase Cγ (PLCγ), SRC family kinases, transforming growth factor beta (TGFβ), and signal transducers and activators of transcription (STATs) [44,45,46]. The presence of the decorin coating on the aligned collagen fibrils led to a decreased levels of PDGFRβ phosphorylation. This result appears to be consistent with reports that decorin [28] and a decorin mimic (DS-SILY20) [47] can attenuate the phosphorylation of the PDGF receptor in human vascular smooth muscle cells. Similarly, it has been reported that decorin affects PDGFR signaling in hepatocarcinogenesis [13]. To our knowledge, our study is the first report to show that decorin modulates PDGFRβ phosphorylation in corneal keratocytes. At this time, it is unclear whether decorin is reducing PDGFRβ phosphorylation by sequestering PDGF locally, as suggested by others [28,47], or binding to and interfering with PDGFRβ activity in a manner similar to its binding and attenuation of the activity of Insulin-like growth factor receptor I (IGF-IR) [48]. In addition, the identity of the downstream intracellular signaling molecules that maybe affected by the reduction in PDGFRβ phosphorylation caused by the decorin coating are unknown and under investigation.

## 4. Materials and Methods

### 4.1. Micropatterning of Aligned Collagen Fibrils

Aligned collagen fibril micropatterns were fabricated by perfusing a Type I collagen solution through straight microfluidic channels bonded to glass coverslips at a well-defined shear rate as previously described [22,49]. Briefly, polydimethylsiloxane (PDMS) microfluidic channels (750 μm wide, 60 μm tall) were treated with air plasma for 1 min on high RF (Harrick Plasma, Ithica, NY, USA). The channels were then promptly sealed onto no. 1.5 glass coverslips (Brain Research Laboratories, Waban, MA, USA) previously cleaned with 28% nitric acid for 1 h and functionalized with a 1% dilution of AquaSil (NC3590194; Thermo Fisher, Waltham, MA, USA) in water for 15 s. The channel and coverslip assemblies were placed on a hotplate (Torrey Pines Scientific, Carlsbad, CA, USA) set at 37 °C for 30 min prior to perfusion to assist with bond formation. A collagen solution was then prepared by mixing a ratio of 8 parts of Type I collagen (Advanced BioMatrix, Carlsbad, CA, USA) to 1 part each of 10× minimal essential media (MEM) (Life Tech, Carlsbad, CA, USA) and 0.1 M NaOH, and adding 1× MEM to reach a collagen concentration of 1.6 mg/mL. Solution pH was then adjusted to 7.55 ± 0.05 with additional 0.1 M NaOH. Using a syringe pump (Harvard Apparatus, Holliston, MA, USA), the collagen solution was perfused through the prepared channel assemblies placed on a 37 °C hotplate in a 4 °C cold room, initially at a higher shear rate of 1500 s^−1^ to pre-charge the channel before reducing the shear to 150 s^−1^ and perfusing for 30 min. Perfused collagen was removed with a pipet from channel outlets, and collagen fibrils were washed by briefly withdrawing ultrapure water through the channel to remove any residual collagen solution prior to channel removal.

### 4.2. Decorin Coating of Aligned Collagen Fibril Micropatterns

Some aligned collagen fibril micropatterns were coated with a solution of 100 nM bovine articular decorin (D-8428, Sigma, St. Louis, MO, USA) in 1× phosphate-buffered saline (PBS) as previously described [10]. Briefly, following the deposition of collagen fibrils in a PDMS microchannel and washing by withdrawal of water through the channel, the 100 nM decorin solution was withdrawn into the channel onto the collagen fibrils and placed into an incubator at 37 °C for 1 h. The collagen fibrils were once again washed by withdrawal of water before removing the PDMS channel, and dried on a hotplate set to 40 °C before use.

### 4.3. Preparation of Aligned Collagen Fibril Micropatterns for Timelapse Cell Culture and Imaging Experiments

For timelapse experiments, aligned collagen fibril micropatterns deposited on glass coverslips were fixed within a one-well plate and bonded to a PDMS ring surrounding the micropattern to facilitate cell culture and enable high-resolution microscopic imaging. Briefly, six 32 mm diameter circular holes were bored out of one-well plates in alignment with the configuration of a standard six-well plate (see Supplemental Figure S3 for a schematic representation of plate assembly). PDMS rings (15 mm inner diameter, 30 mm outer diameter) were then plasma treated for 1 min on high RF and bonded on top of aligned collagen fibril micropatterns deposited on 40 × 40 mm Aquasil-treated glass coverslips. PDMS ring–glass coverslip assemblies were then inserted into the holes in the one-well plate with the glass coverslips flush with the underside of the one-well plate. The entire assembly was then inverted, and the edges of the six glass coverslips were fixed to the plate bottom with epoxy glue and allowed to dry overnight. Prior to cell culture experiments, one-well plates were sterilized with UV for one hour.

### 4.4. Corneal Cell Culture

Primary corneal keratocytes were isolated from New Zealand white rabbit eyeballs (Pel-Freez, Rogers, AR, USA) as previously described [21,49]. Cells were then incubated at 5% CO_2_ and 37 °C with basal serum-free (SF) media constituted of Dulbecco’s Modified Eagle’s (DMEM) media supplemented with 100 μM nonessential amino acids (Invitrogen, Gibco, Waltham, MA, USA), 1% RPMI vitamin mix (Invitrogen, Gibco), and 1% penicillin streptomycin (Invitrogen, Gibco) for 4 days before use. For cell culture experiments, keratocytes were then seeded onto PDMS ring–glass coverslip substrates in 2 mL of SF media at either 20,000 cell/mL or 60,000 cell/mL (12,750 cell/cm^2^ and 38,200 cell/cm^2^, respectively). In some culture wells, PDGF-BB (PHG0045, Fisher Scientific, Waltham, MA, USA) was added to the culture medium at 50 ng/mL.

### 4.5. Freeze Injury

To investigate corneal keratocyte PDGF-BB-induced migration in the presence of a cell density gradient on the aligned collagen fibril micropatterns, we used a freeze injury assay as previously described [21,50]. Briefly, corneal keratocytes were seeded in serum-free (SF) media on aligned collagen fibrils with or without the decorin coating at a density of 60,000 cells/mL (density per unit area) in PDMS ring cell culture wells situated in a multi-well plate. Following 24 h of culture to enable cell attachment, culture media was removed gently with a pipette and a liquid nitrogen-cooled aluminum rod (~1.5 mm in diameter) was held flush with the bottom of the glass coverslip beneath the central region of the aligned collagen fibril micropattern for 15 s to create a decellularized region without damaging the underlying collagen fibrils. Immediately after the freeze injury, substrates were briefly washed with 1 mL SF of media to remove detached cells and inspected under a 10× phase microscope to confirm cell detachment from the wound region. The substrates were then washed once more with 1 mL of serum free media, before 2 mL of either SF media or SF media supplemented with 50 ng/mL of PDGF-BB was added to the wells. The multi-well plate was then placed into a stage incubator (PeCon GmbH, Erbach, Germany) and cultured for 120 h at 5% CO_2_ and 37 °C. Images of the wound regions and adjacent aligned collagen fibrils were captured in each well with phase-contrast microscopy using a Zeiss Axio Observer Z1 inverted microscope (Carl Zeiss, Oberkochen, Germany) with a 10× objective (NA = 0.3) and an Orca Flash 4.0 monochrome CMOS camera (Hamamatsu Photonics, Hamamatsu, Shizuoka, Japan).

### 4.6. Immunofluorescent Staining and Imaging

Upon fixation with 3% paraformaldehyde for 15 min at experiment end points, corneal cells were washed twice with 1× PBS for 10 min and then permeabilized with 0.05% Triton X-100 for 20 min and washed again. Following three washes with 1× PBS, cells were then incubated with Alexa Fluor 488 or 647 conjugated phalloidin (1:400, A12379, A22287, Invitrogen) and DAPI (1:1000) in 2 wt% BSA for 1 h at 37 °C. Cells were then washed and imaged using a Zeiss Axio Observer Z1 inverted microscope with a 20× plan-apochromatic objective (NA = 0.75).

For immunofluorescent labeling of phosphorylated PDGF Receptor β (pPDGFRβ), cells were fixed with 3% paraformaldehyde for 15 min, permeabilized with 0.01% Triton X-100 for 20 min, and blocked overnight with 2 wt% BSA at 4 °C, with washes of 1× PBS between steps. Cells were then incubated with pPDGFRβ (1:50, sc365464, Santa Cruz Biotechnology, Dallas, TX, USA) at 4 °C overnight on a shaker set to approximately 80 RPM. Following washes with 1× PBS, goat secondary antibody against mouse (1:2000, A32723, Invitrogen) was incubated at room temperature for 1 h and washed before imaging with a 63× plan-apocromat oil objective (NA = 1.40) or a 40× plan-neofluar objective (NA = 0.75).

### 4.7. Cell Morphology and Alignment Quantification

Measurement of cell morphology was acquired using ImageJ (NIH; Bethesda, MD, USA; version 1.54f) from the phalloidin-stained images of the cell actin cytoskeleton and DAPI-stained nuclei as previously described [10]. Briefly, fluorescent images were converted to 8-bit and then binary using the set threshold feature. The “wand (tracing) tool” was then used to select and measure individual cells and nuclei for values including projected cell area, Feret’s diameter, aspect ratio, and angle. Cell nuclei orientation index (OI) was calculated using nuclei angles as previously described, with a value of 1 indicating alignment and −1 indicating perpendicular alignment of the nuclei with respect to the aligned collagen fibrils. Keratocyte actin alignment was determined by quantifying the image content percentage aligned with each radial angle using the Directionality plugin in ImageJ. Orientation of the keratocyte actin images was then calculated using the following equations:

$${OI}_{Keratocytes}\left(\theta \right)=\left\{2\langle \cos^{2}\theta \rangle -1\right\}$$

where $\cos^{2}\theta =\frac{\int_{-90}^{90}I\left(\alpha \right)\left\{\cos^{2}\alpha -\theta \right\}dx}{\int_{-90}^{90}I\left(\alpha \right)dx}$ and θ=collagen fibril direction.

### 4.8. Cell Tracking and Directionality Ratio Calculation

To measure cell movement and direction from timelapse experiments, individual cell positions over time were recorded with Image-pro Premier (Media Cybernetics; Rockville, MD, USA; version 9.2) software by selecting the center of the cell nucleus with the “motion tracking” feature. For each experiment, 10–20 cells were measured per experimental group. To quantify persistence or “straightness” of cell migration trajectories, the directionality ratio was calculated by dividing the displacement between the end and starting point of a cell by the total length of its trajectory [51]. The directionality ratio approaches 1 for straight cell trajectories and approaches 0 for cells that change their direction of migration often. Directionality ratio was calculated at the same end time point for all cells analyzed.

### 4.9. Phosphorylated PDGFR-β Quantification

To determine if the influence of the decorin coating on NRK response to PDGF-BB was directly associated with the PDGF-signaling pathway, immunofluorescent staining and analysis of phosphorylated PDGF Receptor β (pPDGFRβ) was performed in the following experiments. NRK were seeded at 20,000 cell/mL (12,750 cell/cm^2^) in 2 mL of serum-free media onto aligned collagen fibrils with or without the decorin coating. Following 24 h of culture at 37 °C and 5% CO_2_, 1 mL of media was removed from each culture well and replaced with 1 mL of either fresh media or media supplemented with PDGF-BB to reach a concentration of 50 ng/mL once added. Cells were cultured for 15 min before fixation and immunofluorescent labeling as described. The amount of pPDGFRβ following treatment with PDGF-BB was quantified by measurement of the mean grey values in 8-bit images of the pPDGFRβ staining within individual cell bodies as defined by their stained actin cytoskeleton. For each sample across 4 experimental replicates, 40 cells from 10 images were analyzed for a total of 160 cells analyzed per treatment group. Staining conditions and imaging settings were maintained between samples and across experimental replicates.

### 4.10. Statistics

For each data plot, 3 or more experimental replicates were analyzed and are represented as mean ± standard deviation. Statistical analysis of data was performed in Prism 8 (GraphPad, San Diego, CA, USA) using one or two-way ANOVA and Tukey’s post hoc test. All *p*-values are specified in figure captions.

## 5. Conclusions

While decorin has been suggested as a potential promising anti-fibrotic agent due to its inhibition of TGFβ in several tissue types, there is still much unknown about how decorin interacts with other key components of the cornea relevant to the wound-healing process. For example, although some studies have shown that treatment by decorin in media elicits a change in cell behavior [5,28], others have observed that decorin must be immobilized to the ECM to inhibit growth factor activity [16,20]. Since the migration of PDGF-BB-stimulated keratocytes in the cornea is highly dependent on the unique organization of the corneal ECM, it is likely that the inhibition of PDGF signaling in the cornea by decorin is also dependent on this organization. For the first time, this study demonstrates the inhibition of PDGF signaling in corneal keratocytes on aligned collagen fibrils mimicking the native corneal stroma ECM topography. Keratocyte responses to PDGF-BB, including motility, alignment, wound closure, migration persistence, bipolarization, and PDGFRβ phosphorylation, were shown to be reduced. Interestingly, while PDGF-stimulated migration on decorin-coated aligned collagen fibrils both with and without the freeze injury was decreased, the drop in cell movement was much lower than the dramatic slowing of cell movement without the injury. Coupled with the loss of persistent cell migration on the decorin coating, this discrepancy may point to a loss of guided migration typically observed in keratocytes stimulated by PDGF-BB on aligned collagen fibrils or in the cornea. While additional studies are needed to better understand decorin’s role in corneal wound healing and PDGF signaling specifically, these data show that decorin can impede migration during wound healing. Thus, the effects on PDGF signaling should be an important consideration in the continued development and use of decorin in anti-fibrotic treatment.

## Figures and Tables

**Figure 1:**
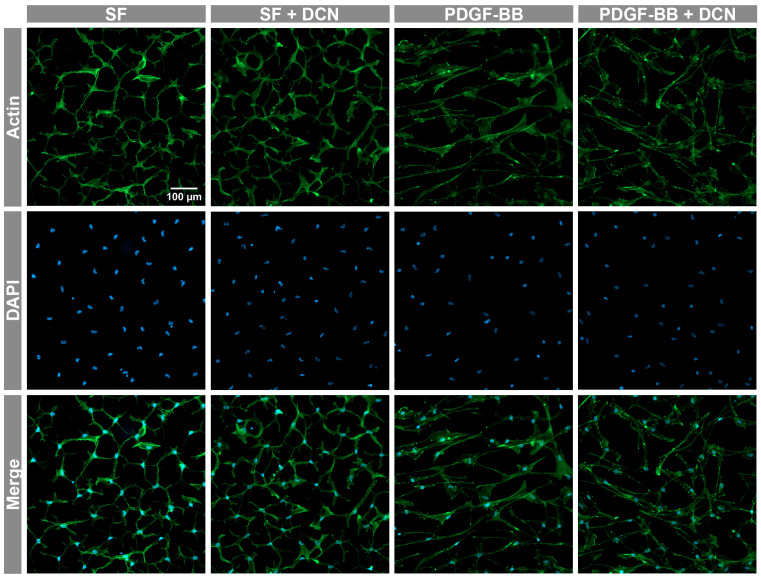
PDGF-stimulated corneal keratocyte alignment is reduced on decorin-coated aligned collagen fibrils. Immunofluorescent images of cell actin cytoskeleton (Phalloidin, green) and nuclei (DAPI, blue) of primary rabbit keratocytes cultured for 5 days on substrates with aligned collagen fibrils with and without a decorin (DCN) coating in serum free (SF) media or media containing PDGF-BB (50 ng/mL). The aligned collagen fibril orientation was horizontal (i.e., left to right) for all four substrates.

**Figure 2:**
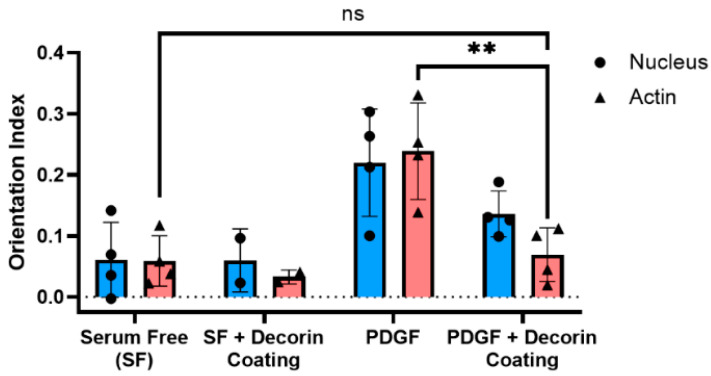
PDGF-stimulated actin cytoskeleton alignment is reduced on aligned collagen fibrils in the presence of a decorin coating. Plot of the mean ± s.d. of the orientation index (OI) of actin and DAPI immunostaining cell images from 4 experimental replicates. Nucleus OI was calculated using the nuclei major axis angles while actin OI was calculated using radial angle contributions of all image features obtained using the Directionality plugin in ImageJ, with a value of 1 indicating parallel alignment with the fibrils, and −1 indicating perpendicular alignment. A two-way ANOVA with a Tukey post-hoc test was used to determine significance between groups (** *p* < 0.01).

**Figure 3:**
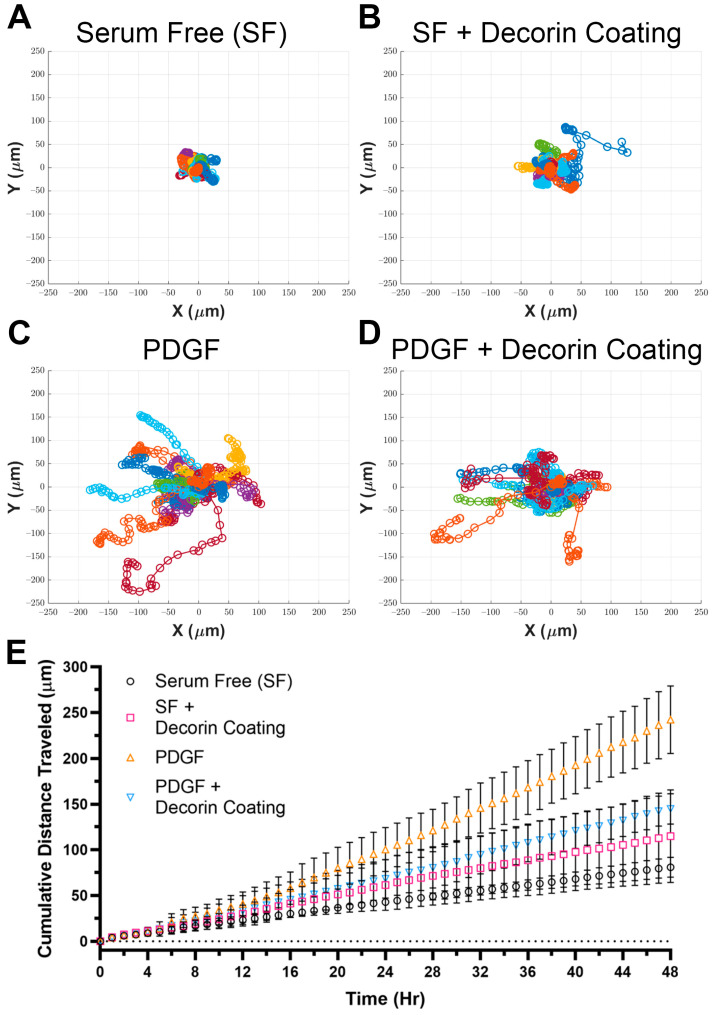
Effect of decorin coating on PDGF-mediated keratocyte motility. Cell tracking profiles of 30 representative cells cultured in (**A**) serum-free media on aligned collagen fibrils, (**B**) serum free media on decorin coated aligned collagen fibrils, (**C**) media containing 50 ng/mL PDGF-BB on aligned collagen fibrils, and (**D**) media containing 50 ng/mL PDGF-BB on decorin coated aligned collagen fibrils. Each color represents the trajectory of an individual cell. (**E**) plot of mean ± s.d. accumulated distance traveled from 90 cells over 48 h from *n* = 3 experimental replicates.

**Figure 4:**
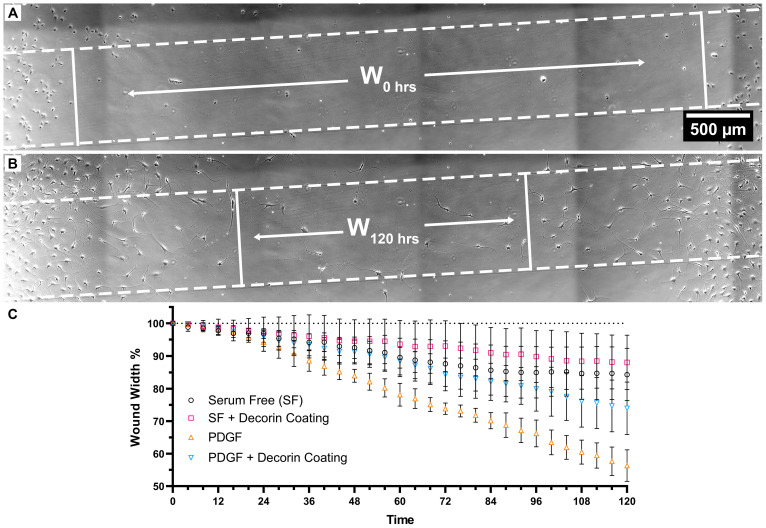
Decorin coating reduces PDGF-mediated wound closure in freeze injury assay. Phase contrast images of freeze injury wound region W at (**A**) 0 h and (**B**) 120 h following the initial injury. Dotted lines indicate aligned collagen fibril micropattern boundaries. (**C**) Plot of mean ± s.d. wound width relative to initial width over time for *n* = 4 experimental replicates.

**Figure 5:**
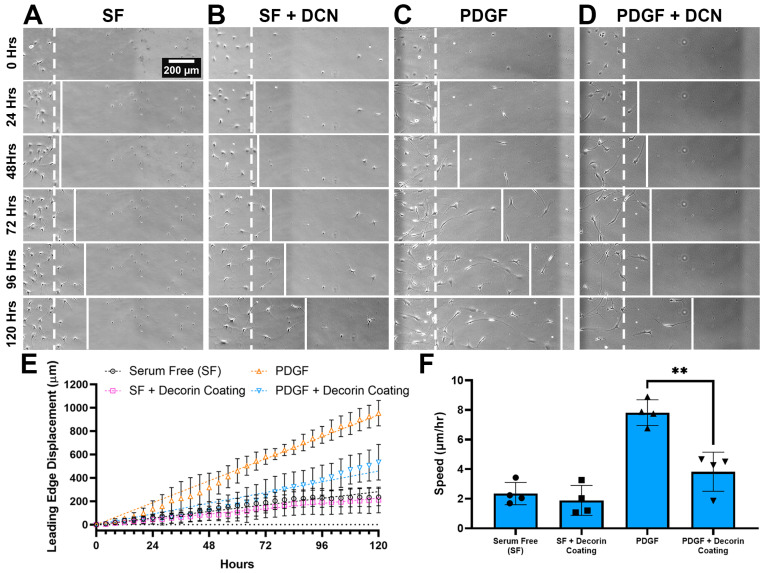
Leading edge displacement of PDGF-treated NRK is reduced on decorin-coated aligned collagen fibrils. (**A**–**D**) Representative phase contrast images of the leading edge of NRK cells migrating into a freeze injury in serum free (SF) media or media supplemented with PDGF-BB on substrates coated with aligned collagen fibrils with and without the decorin coating. The dashed white line indicates the initial wound border, while the solid white line indicates the final wound border. (**E**) Quantification of the wound leading edge displacement over time. (**F**) Plot of the wound leading edge speed. Each data point in panels (**E**,**F**) represents the mean ± s.d. from *n* = 4 replicates (** *p* < 0.01).

**Figure 6:**
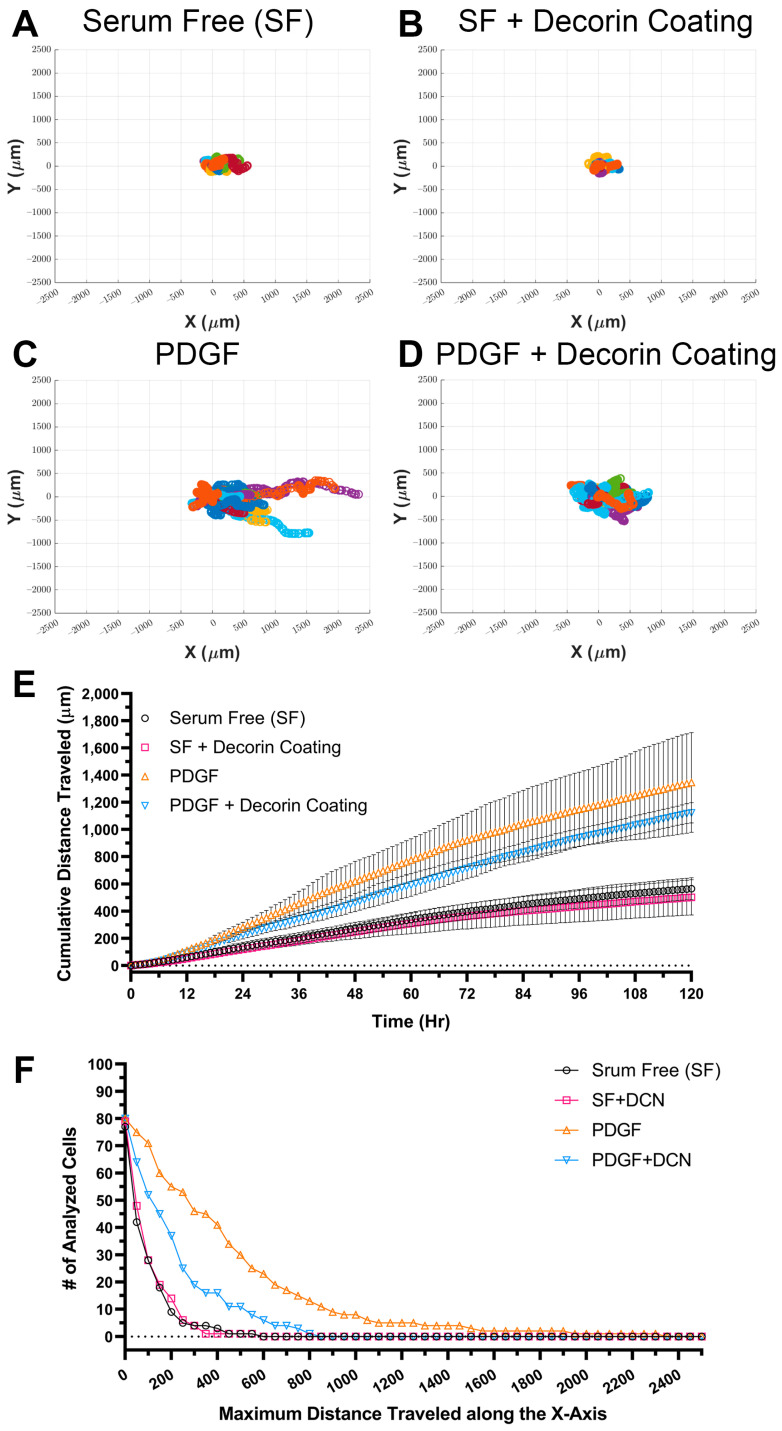
PDGF-mediated individual cell migration and wound penetration is reduced on decorin-coated aligend collagen fibril micropatterns. (**A**–**D**) Plot of 30 representative tracks of cells migrating into a freeze injury when cells were cultured on uncoated or decorin-coated aligned collagen fibrils in either serum-free conditions or in media supplemented with 10 ng/mL PDGF-BB. Each color represents the trajectory of an individual cell. (**E**) Plot of mean ± s.d. accumulated distance traveled by NRK cells over 120 h after the freeze injury. (**F**) Distribution of the distance traveled by cells from the initial wound edge towards the decellularized wound region under the different culture conditions following a freeze injury.

**Figure 7:**
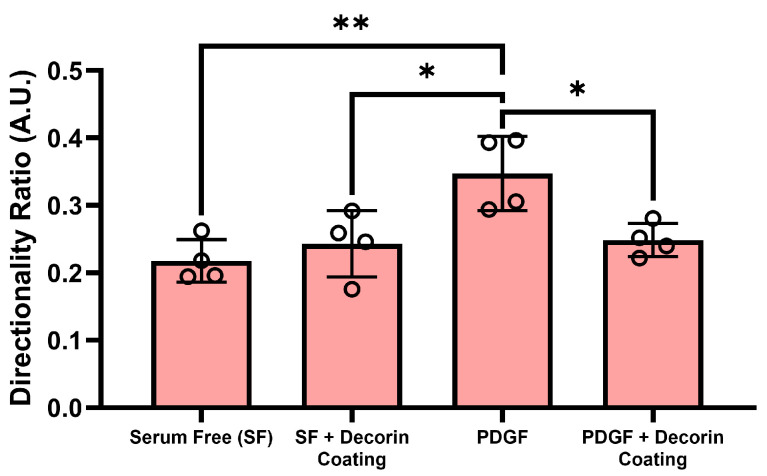
PDGF-mediated increase in migration directionality ratio on the aligned collagen fibrils is lost in the presence of the decorin coating. Plot of mean ± s.d. of the directionality ratio at t = 120 h after a a freeze injury from *n* = 4 experimental replicates and 20 cells per replicate (* *p* < 0.05. ** *p* < 0.01).

**Figure 8:**
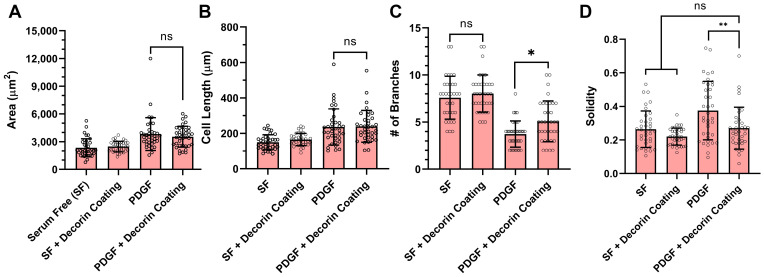
Effect of decorin coating on keratocyte morphology. Plots of cell (**A**) projected area, (**B**) length, (**C**) number of branches, and (**D**) solidity of cells migrating towards a freeze injury when cultured in serum free (SF) media or media supplemented with PDGF-BB conditions on substrates coated with aligned collagen fibrils with and without the decorin coating. Each data point plot represents the value from one cell. The bar graphs and error bars represent mean ± s.d. (from *n* = 4 experimental replicates) (* *p* < 0.05. ** *p* < 0.01).

**Figure 9:**
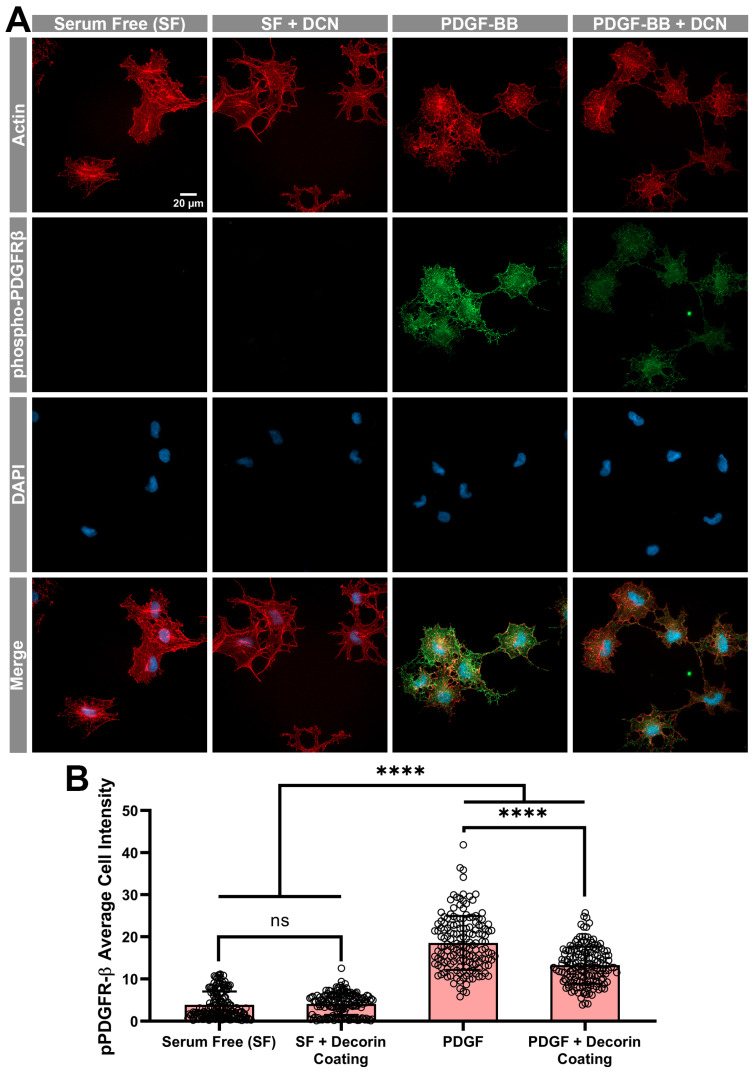
Effect of Decorin Coating on PDGFR-β Phosphorylation. (**A**) Representative fluorescent images of keratocytes cultured on uncoated and decorin-coated aligned collagen fibrils in serum-free media or media containing 50 ng/mL PDGF-BB. Cells were seeded and cultured on the different substrates for 24 h before exposing the cells to swapping the media to expose the cells to serum-free media or media containing PDGF-BB or for 15 min. Cells were subsequently fixed, permeabilized and stained before fluorescent images were taken. Cells were stained for F-actin (red), phosphorylated PDGFR-β (green), and nuclei (blue), scale = 20 µm. (**B**) Plot of the mean ± s.d. average cell PDGFR-β fluorescence in untreated (serum free media only) cells, or cell treated for 15 min with 50 ng/mL PDGF-BB (**** *p* < 0.0001).

## Data Availability

Data are contained within the article and Supplementary Materials.

## Supplementary Materials

The following supporting information can be downloaded at: https://www.mdpi.com/article/10.3390/ijms27156829/s1.
