# Supplementary material for: PDGF-Stimulated Corneal Keratocyte Motility and Migration Patterns on Aligned Collagen Fibrils Are Inhibited by Decorin
Source: Int J Mol Sci. 2026 Jul 30;27(15):6829. doi: 10.3390/ijms27156829 (PMC13467390; doi:10.3390/ijms27156829)
Supplement: Supplementary file 1 [file ijms-27-06829-s001.zip › Tjahjono-IJMS2026 - Supplementary Data.pdf]

Supplementary Data

for

Article

PDGF Stimulated Corneal Keratocyte Motility and Migration Patterns on Aligned Collagen Fibrils Are Inhibited by Decorin

Nathaniel S. Tjahjono[1], Divya Subramanian[1], Tarik Z. Shihabeddin[1], Nishtha Tyagi[1], Marya Zlotnikova[1], Miguel Miron-Mendoza[2], Victor D. Varner[1,3], W. Matthew Petroll[2,3], and David W. Schmidtke[1,3*]

[1] Department of Bioengineering, University of Texas at Dallas, Richardson, TX 75080, USA nathaniel.tjahjono@utdallas.edu (N.S.T.); divya.subramanian@utdallas.edu (D.S.), tarik.shihabeddin@utdallas.edu (T.Z.S.); nishtha.tyagi@utdallas.edu (N.T.); marya.zlotnikova@utdallas.edu (M.Z.); vdv@utdallas.edu (V.D.V.)

[2] Department of Ophthalmology, University of Texas Southwestern Medical Center, Dallas, TX 75090, USA miguel.miron@utsouthwestern.edu (M.M.-M.); matthew.petroll@utsouthwestern.edu (W.M.P.)

[3] Department of Biomedical Engineering, University of Texas Southwestern Medical Center, Dallas, TX 75390

* Correspondence: David.Schmidtke@utdallas.edu;

Supplementary Figures

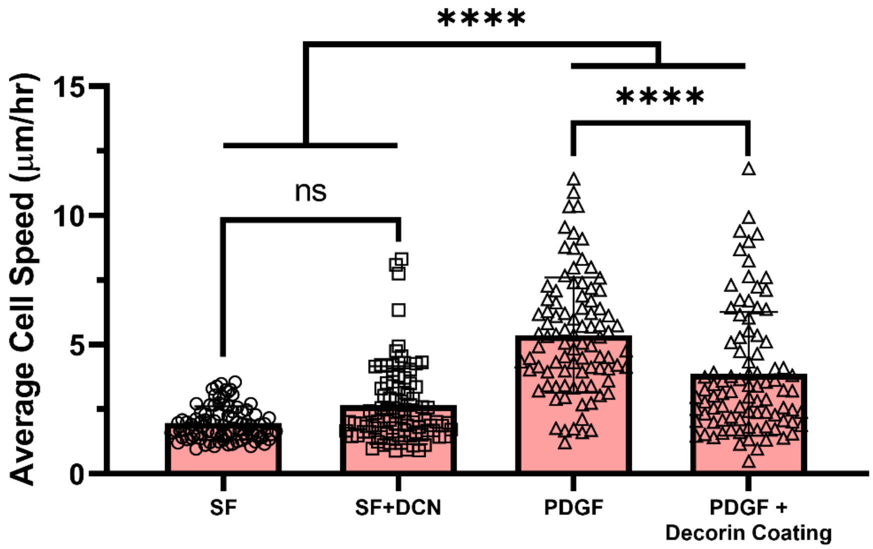

Figure S1. Average speed of cells after seeding on aligned collagen fibril substrates for 48 hours.
*Plot of the mean ± s.d. cell speed for 90 cells from N = 3 experimental replicates (**** p < 0.0001).*

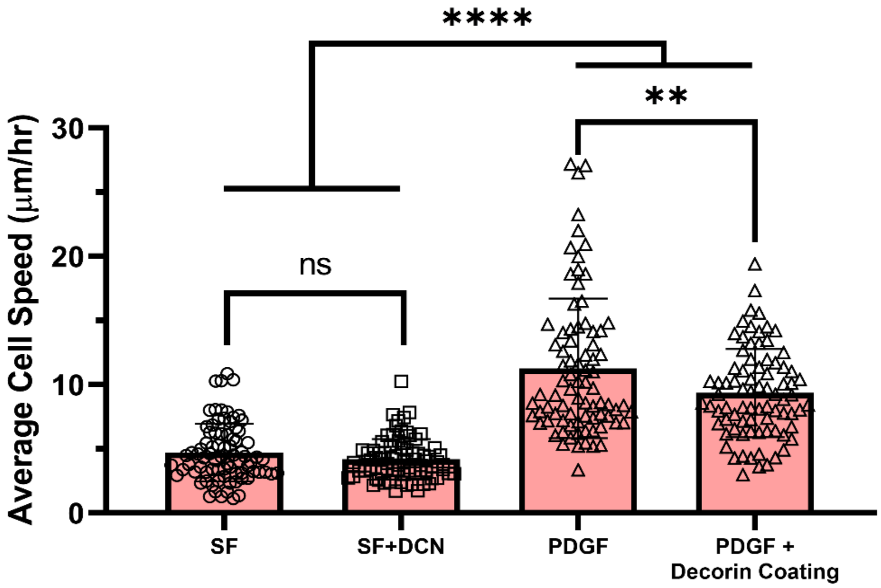

Figure S2. Average speed of cells over 120 hours following a freeze injury on aligned collagen fibril substrates. *Plot of mean ± s.d. cell speed for 80 cells from N = 4 experimental replicates (** $p < 0.01$, **** $p < 0.0001$).*

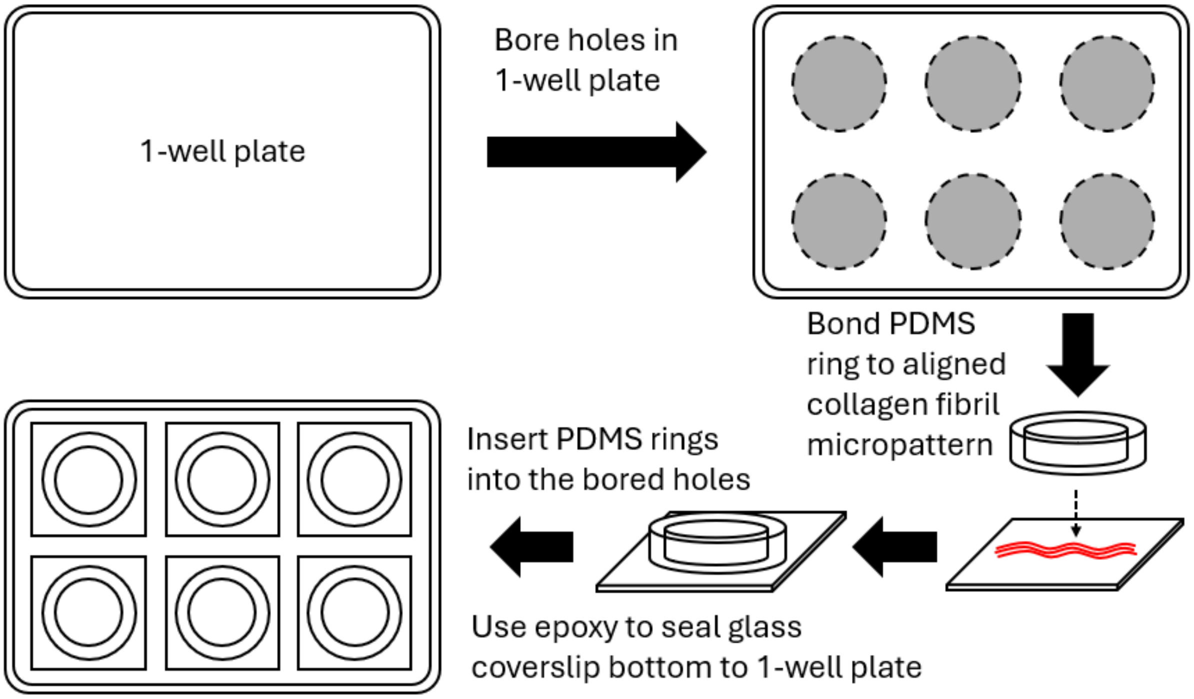

Figure S3. Schematic of 1-well plate modification to accommodate 6 micropatterns of aligned collagen fibrils for timelapse imaging.
